# Histopathological Spectrum in FIT‐Positive Patients With Abnormal Colonoscopy Findings at a Tertiary Medical Center in Lebanon: A Retrospective Observational Study

**DOI:** 10.1002/hsr2.73409

**Published:** 2026-10-18

**Authors:** Fatima El Houjeiry, Walid Nasreddine, Alissar El Sibaii, Hala Thaalibi, Farah Singer, Bilal Azakir

**Affiliations:** ^1^ Faculty of Medicine Beirut Arab University Beirut Lebanon; ^2^ Department of Internal Medicine Makassed General Hospital Beirut Lebanon

**Keywords:** clinical indications, colonoscopy, colorectal cancer screening, fecal occult blood test (FIT), histopathology, neoplastic polyps

## Abstract

**Background and Aims:**

Colorectal cancer is a leading cause of cancer‐related morbidity and mortality, and the prognosis significantly depends on the diagnostic stage. Various screening initiatives have been established, among which the fecal immunochemical test (FIT) (FITFIT) which is prominent for its affordability, non‐invasive approach, and wide acceptance. This study aimed to describe the spectrum of histopathological findings among patients with positive fecal occult blood test and abnormal colonoscopy findings, and to identify associated risk factors.

**Methods:**

This retrospective observational study included 172 adults with documented positive FIT and abnormal colonoscopy findings at Makassed General Hospital from January 2024 to December 2025. Statistical analysis was conducted using SPSS version 25, and associations were assessed using chi‐square or Fisher's exact tests, with significance defined as *p* < 0.05.

**Results:**

A total of 172 patients were included, with a mean age of 63.88 ± 11.79 years, and 55.2% were female. The most frequently recorded indications for colonoscopy were anemia (39%) and screening (37.2%). Polyps were the most common colonoscopy finding. Histopathological examination demonstrated that neoplastic polyps represented the predominant diagnosis (65.1%), followed by non‐neoplastic lesions (24.4%) and malignancy (10.5%). Patients aged 45 years and older demonstrated a higher prevalence of polyps on colonoscopy; however, the difference did not reach statistical significance. No significant association was observed between gender and colonoscopy or histopathological findings. Abdominal pain and diarrhea were both significantly associated with non‐neoplastic histopathological lesions.

**Conclusion:**

Among patients with positive FIT and abnormal colonoscopy findings, neoplastic polyps rather than malignancy represented the predominant histopathological findings, supporting the clinical utility of FIT in detecting clinically important colorectal lesions at earlier stages. In addition, variability in the histopathology depending on the clinical presentation may contribute to clinical assessment and risk stratification. Larger prospective studies are needed to determine predictors of advanced colorectal histopathology.

## 1 Introduction

Colorectal cancer (CRC) remains a major contributor to morbidity and mortality worldwide and continues to represent a significant public health concern [1, 2, 3]. Early detection through screening is essential for reducing disease burden and improving survival outcomes [1, 2, 3, 4]. Fecal occult blood testing (FOBT/FIT) is widely used in colorectal cancer screening programs because of its non‐invasive nature, accessibility, and cost‐effectiveness [4, 5, 6, 7].

Patients with positive FOBT/FIT results are generally referred for colonoscopy, which allows direct visualization of the colonic mucosa and enables tissue sampling for definitive histopathological diagnosis [4, 6, 7, 8]. Although positive stool‐based screening tests effectively identify individuals who require further diagnostic evaluation, colonoscopy findings may vary considerably, ranging from non‐neoplastic and inflammatory lesions to adenomatous polyps and invasive colorectal malignancies [6, 7, 8]. Consequently, positive FOBT/FIT results alone cannot distinguish the underlying pathological condition and must be interpreted alongside colonoscopy and histopathological findings [4, 5, 6, 7].

Clinical presentation may additionally provide insight into the underlying colorectal pathology. Symptoms such as anemia, abdominal pain, altered bowel habits, diarrhea, and rectal bleeding are common indications for colonoscopy and may correlate with specific histopathological outcomes [6, 9, 10, 11]. Understanding the association between clinical manifestations and pathological findings in patients with positive FOBT/FIT results may improve diagnostic evaluation and support more informed clinical decision‐making [6, 9, 10, 11].

Despite the widespread use of FOBT/FIT in colorectal cancer screening initiatives, data regarding the spectrum of histopathological findings among patients with positive stool‐based screening tests remain limited, particularly in Lebanon and similar regional healthcare settings [6, 7, 8]. Existing evidence is largely derived from international populations, while local real‐world data describing colonoscopic and histopathological outcomes among FIT‐positive patients are scarce.

Fecal occult blood testing is widely implemented in colorectal cancer screening programs, yet there is limited evidence identifying the spectrum of histopathological findings among FIT‐positive patients in Lebanon and the Eastern Mediterranean region, as most of the information is provided by studies conducted among European and Asian populations. These studies involved different disease characteristics, screening practices, and referral pathways. Moreover, few studies have simultaneously identified histopathological diagnosis and colonoscopy findings in relation to sociodemographic characteristics of the population and the clinical indication in their clinical practice. Thus, this study aimed to provide local real‐world evidence within regional healthcare settings, providing information in context‐specific colorectal cancer screening practices by characterizing the histopathological spectrum among FIT‐positive patients with abnormal colonoscopy findings in a Lebanese tertiary medical center, and to examine their associated risk factors and indications for colonoscopy.

## 2 Methods

### 2.1 Study Design and Setting

This retrospective observational study was conducted at Makassed General Hospital through review of electronic medical records of patients evaluated between January 1, 2024, and December 31, 2025. The study aimed to characterize the histopathological findings among patients with positive FITFIT results who underwent colonoscopy with abnormal findings.

### 2.2 Study Population

Adult patients aged 18 years and older with a documented positive FIT who subsequently underwent colonoscopy demonstrating abnormal findings and had available histopathological biopsy results were eligible for inclusion in the study. Patients were excluded if colonoscopy findings were normal despite a positive FIT, if medical records or histopathological reports were incomplete or unavailable, or if there was a prior documented diagnosis of colorectal cancer. All patients included in the study had a positive fecal immunochemical test (FIT) before undergoing colonoscopy.

### 2.3 Data Collection

A retrospective review of the hospital electronic records system was conducted to identify adult patients with positive fecal occult blood test who underwent colonoscopy with abnormal findings and available histopathological results. FIT was performed as part of the evaluation of symptoms or clinical conditions, or as screening for colorectal cancer. Data were extracted by the investigators using a data collection sheet. Data on the histopathological diagnoses were extracted from the pathology reports. Any patients with missing key data were excluded. Extracted information included demographic characteristics, clinical indications for colonoscopy, colonoscopic findings, and histopathological results.

### 2.4 Variables and Definitions

#### 2.4.1 Demographic Variables

Demographic data included age and gender for all included patients.

#### 2.4.2 Clinical Variables

Clinical indications for colonoscopy were categorized into screening, anemia, abdominal pain, diarrhea, constipation, and other indications, including weight loss and bloating.

#### 2.4.3 Colonoscopic Findings

Abnormal colonoscopic findings were categorized as polyps, masses, hemorrhoids, inflammatory lesions (colitis and ileitis), diverticulosis, and other abnormalities such as ulcers and arteriovenous malformations.

#### 2.4.4 Histopathological Findings

Histopathological findings were classified into three principal categories:

1. Neoplastic polyps, including tubular adenoma, tubulovillous adenoma, villous adenoma, low‐grade dysplasia, and high‐grade dysplasia.
2. Non‐neoplastic lesions, including:
  - Non‐neoplastic polyps, defined as lesions without dysplasia or malignant potential, such as hyperplastic and inflammatory polyps
  - Inflammatory bowel disease (IBD)‐related colitis
  - Non‐IBD colitis

3. Malignancy, defined as invasive adenocarcinoma.

#### 2.4.5 Operational Definition

An abnormal colonoscopy was defined as any colonoscopic lesion requiring biopsy, including polyps, masses, ulcers, or inflammatory lesions.

### 2.5 Sample Size

Because this was a retrospective observational study, all patients meeting the inclusion criteria during the study period were included in the analysis. Therefore, no formal sample size calculation was performed.

### 2.6 Statistical Analysis

Data analysis was performed using the IBM SPSS Statistics for Windows, Version 25. (IBM Corp., Armonk, NY, USA). Continuous variables were computed as mean ± standard deviation, while categorical variables were summarized as frequencies and percentages and compared by using the *χ* ^2^ test or Fisher's exact test, as appropriate. To determine factors independently associated with neoplastic histopathological findings, a multivariable binary logistic regression analysis was performed, including age, gender, abdominal pain, and diarrhea. Adjusted odds ratios (aORs) with 95% confidence intervals (CIs) were reported. All statistical tests were two‐sided and a *p* value of less than 0.05 was considered statistically significant.

### 2.7 Ethical Considerations

The study protocol was approved by the Institutional Review Board (IRB 031224k) of Makassed General Hospital. Patient confidentiality was maintained throughout the study by anonymizing and coding all collected data. The requirement for informed consent was waived by the IRB due to the retrospective design of the study. The study was conducted in accordance with the Strengthening the Reporting of Observational Studies in Epidemiology (STROBE) guidelines.

## 3 Results

### 3.1 Baseline Characteristics

A total of 172 patients were included in the study. Of these patients, 44.8% were men, whereas 55.2% were female, with a mean age of 63.88 years (Table 1). Anemia was the most common indication for colonoscopy, reported in 39% of patients, followed by screening indications in 37.2%. Other indications included diarrhea (11.6%), abdominal pain (8.7%), constipation (1.2%), and miscellaneous indications (4.7%) (Table 1).

**Table 1:** Demographic characteristics and clinical indications for colonoscopy of the study population.

Variable	Value
Age (years), mean ± SD	63.88 ± 11.79
Gender, *n* (%)	
Male	77 (44.8)
Female	95 (55.2)
Clinical indication for colonoscopy, *n* (%)	
Screening	64 (37.2)
Anemia	67 (39.0)
Abdominal pain	15 (8.7)
Diarrhea	20 (11.6)
Constipation	2 (1.2)
Other^a^	8 (4.7)

*Note:* Clinical indications were not mutually exclusive, and some patients had more than one indication for colonoscopy.

a Other indications included weight loss and bloating.

### 3.2 Colonoscopy and Histopathology Results

Colonoscopy demonstrated a broad spectrum of abnormal findings. Polyps were the most frequently identified colonoscopic finding. Other abnormalities recorded included hemorrhoids, inflammatory lesions, masses, diverticulosis, and miscellaneous lesions (Figure 1).

**Figure 1:**
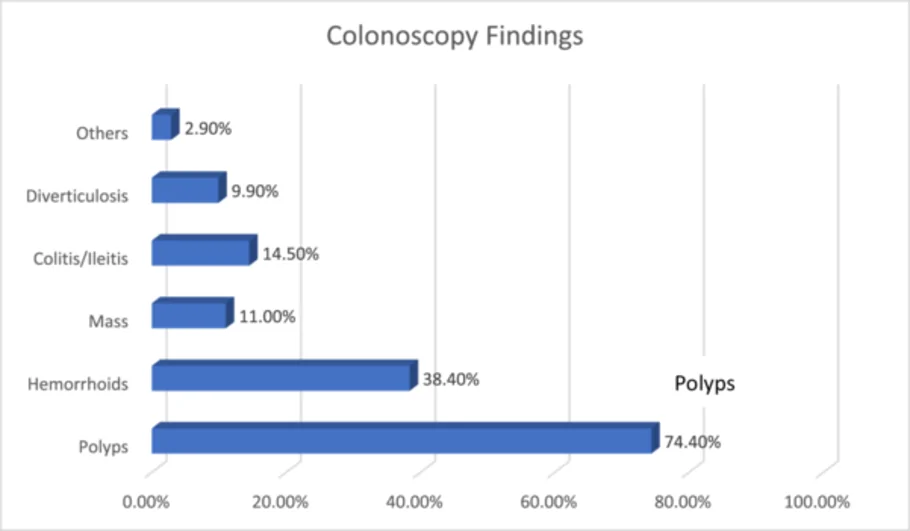
Colonoscopic findings among the study population.

Histopathological examination demonstrated that neoplastic polyps constituted the predominant pathological finding, accounting for 65.1% of cases. Non‐neoplastic lesions represented 24.4% of cases, whereas malignant lesions were identified in 10.5% of the study population (Figure 2).

**Figure 2:**
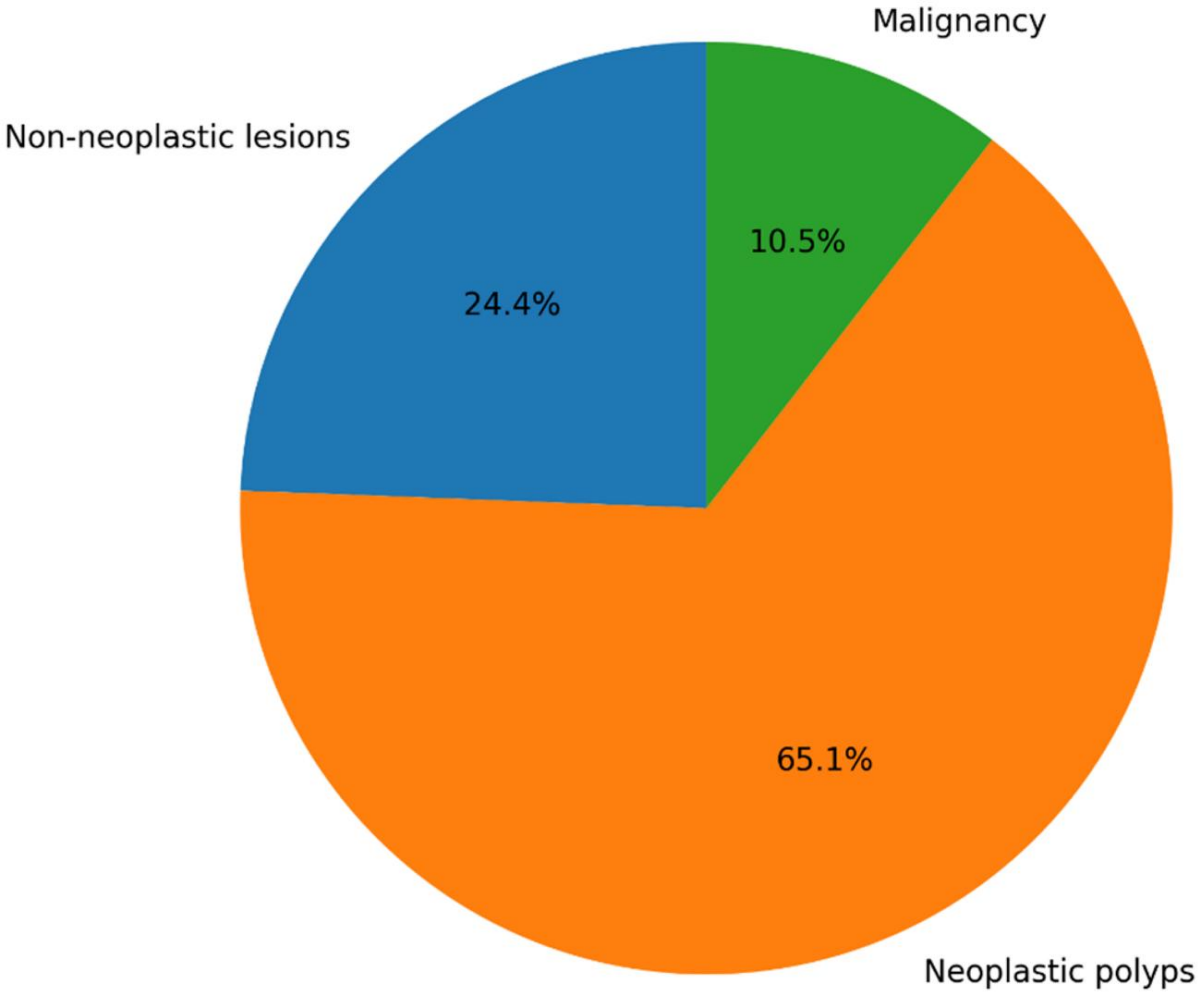
Distribution of histopathological findings among the study population.

### 3.3 Association Between Age and Study Variables

Patients aged 45 years and older demonstrated a higher prevalence of polyps on colonoscopy compared with patients younger than 45 years, yet did not reach statistical significance (76.1% vs. 44.4%, *p *= 0.23).

A significant association was also observed between younger age and the presence of inflammatory lesions (*p *= 0.02) (Table 2).

**Table 2:** Association between age groups, clinical indications, colonoscopic findings, and histopathological findings.

Variable	Age < 45 years (*n* = 9)	≥ 45 Years (*n* = 163)	*p* value
Clinical indication for colonoscopy, *n* (%)			
Screening	1 (11.1)	63 (38.7)	0.15
Anemia	5 (55.6)	62 (38.0)	0.31
Abdominal pain	1 (11.1)	14 (8.6)	0.56
Diarrhea	2 (22.2)	18 (11.0)	0.28
Constipation	0 (0.0)	2 (1.2)	> 0.99
Other indications	0 (0.0)	8 (4.9)	> 0.99
Colonoscopic findings, *n* (%)			
Polyps	5 (55.6%)	124 (76.1)	0.04
Hemorrhoids	4 (44.4)	62 (38.0)	0.73
Masses	0 (0.0)	19 (11.7)	0.6
Inflammatory lesions (colitis/Ileitis)	4 (44.4)	21 (12.9)	0.02
Diverticulosis	1 (11.1)	16 (9.8)	> 0.99
Other lesions	0 (0.0)	5 (3.1)	> 0.99
Histopathological findings, *n* (%)			
IBD colitis	0 (0.0)	10 (6.1)	> 0.99
Non‐IBD colitis	3 (33.3)	23 (14.1)	0.13
Neoplastic polyps	4 (44.4)	108 (66.3)	0.28
Non‐neoplastic polyps	1 (11.1)	14 (8.6)	0.56
Malignancy	0 (0.0)	18 (11.0)	0.60
Other	1 (11.1)	1 (0.6)	0.10

No statistically significant associations were identified between age group and colonoscopy indications or histopathological findings (*p *> 0.05 for all comparisons).

### 3.4 Association Between Gender and Study Variables

No statistically significant differences were observed between male and female patients regarding clinical indications for colonoscopy, colonoscopic findings, or histopathological outcomes (Table 3). Screening and anemia were the most common clinical indications in both groups. Polyps represented the predominant colonoscopic finding among males (77.9%) and females (71.6%), while neoplastic polyps were the most frequent histopathological diagnosis in both sexes. Although males demonstrated slightly higher rates of masses and malignancy, these differences did not reach statistical significance (Table 3).

**Table 3:** Association between gender and clinical, colonoscopic, and histopathological findings.

	Gender		
	Male (77)	Female (95)	*p* value
Indication			
Screening	28 (36.4%)	36 (37.9%)	0.87
Anemia	31 (40.3%)	36 (37.9%)	0.75
Abdominal pain	6 (7.8%)	9 (9.5%)	0.79
Diarrhea	7 (9.1%)	13 (13.7%)	0.47
Constipation	—	2 (2.1%)	0.50
Others	6 (7.8%)	2 (2.1%)	0.14
Colonoscopic findings			
Polyps	60 (77.9%)	68 (71.6%)	0.38
Hemorrhoids	32 (41.6%)	34 (35.8%)	0.52
Masses	11 (14.3%)	8 (8.4%)	0.32
Colitis/ileitis	8 (10.4%)	17 (17.9%)	0.19
Diverticulosis	7 (9.1%)	10 (10.5%)	0.8
Others	1 (1.3%)	4 (4.2%)	0.38
Histopathological findings			
IBD colitis	2 (2.6%)	8 (8.4%)	0.18
Non‐IBD colitis	10 (13%)	16 (16.8%)	0.52
Neoplastic polyps	53 (68.8%)	59 (62.1%)	0.42
Non‐neoplastic polyps	8 (10.4%)	7 (7.4%)	0.59
Malignancy	11 (14.3%)	7 (7.4%)	0.21
Others	1 (1.3%)	1 (1.1%)	> 0.99

### 3.5 Association Between Clinical Indications and Histopathological Outcomes

Patients presenting with abdominal pain or diarrhea were more likely to have non‐neoplastic histopathological lesions than neoplastic lesions or malignancy. No associations were identified for screening, anemia, constipation, or other clinical indications. Although no significant associations were identified for screening or anemia, these were the most frequently recorded clinical indications among patients with neoplastic polyps and malignancy, accounting for 40.2% and 42.0% of cases, respectively. In the malignancy group, anemia represented the most frequent clinical indication (50.0%). Significant associations between clinical indications and histopathological outcomes were observed for abdominal pain (*p *= 0.027) and diarrhea (*p *= 0.004), whereas no significant associations were identified for the remaining clinical indications (Table 4).

**Table 4:** Association between clinical indications and histopathological outcomes.

Clinical indication	Non‐neoplastic lesions, *n* = 42	Neoplastic polyps, *n* = 112	Malignancy, *n* = 18	*p* value
Screening	14 (33.3)	45 (40.2)	5 (27.8)	0.53
Anemia	11 (26.2)	47 (42.0)	9 (50.0)	0.13
Abdominal pain	8 (19.0)	7 (6.3)	0 (0.0)	0.02
Diarrhea	11 (26.2)	7 (6.3)	2 (11.1)	0.004
Constipation	0 (0.0)	1 (0.9)	1 (5.6)	0.25
Other indications	0 (0.0)	6 (5.4)	2 (11.1)	0.1

*Note:* Clinical indications were not mutually exclusive; some patients had more than one indication for colonoscopy. Percentages are column percentages. Non‐neoplastic lesions included non‐neoplastic polyps, inflammatory bowel disease (IBD)‐related colitis, non‐IBD colitis, and other benign histopathological findings. *p* values were calculated using the *χ* ^2^ test or Fisher's exact test, comparing the distribution of histopathological outcomes across patients with and without each clinical indication.

### 3.6 Multivariate Logistic Regression Analysis

To determine factors associated independently with neoplastic histopathological findings, a multivariate logistic regression was performed. Patients with diarrhea (adjusted OR 0.21, 95% CI 0.08–0.58; *p *= 0.002) and abdominal pain (adjusted OR 0.22, 95% CI 0.07–0.71; *p *= 0.011) showed significantly lower odds of neoplastic lesions after adjustment. However, age and gender showed no independent association with neoplastic pathology (Table 5).

**Table 5:** Multivariable logistic regression analysis of factors associated with neoplastic histopathological findings.

Variable	Adjusted OR	95% CI	*p* value
Age (per year)	1.01	0.98–1.04	0.58
Female sex	0.47	0.21–1.03	0.05
Abdominal pain	0.22	0.07–0.71	0.01
Diarrhea	0.21	0.08–0.58	0.002

## 4 Discussion

This retrospective observational study investigated the wide spectrum of histopathological findings in patients with positive fecal occult blood test (FIT) results and abnormal colonoscopy findings at Makassed General Hospital. Neoplastic polyps represented the predominant histopathological finding, whereas malignancy was identified in a smaller yet clinically important proportion of patients. Several associations were also observed between demographic characteristics, clinical indications, and colonoscopic or histopathological findings.

Neoplastic polyps accounted for 65.1% of histopathological findings, whereas colorectal cancer was identified in 10.5% of patients (Figure 2), which is consistent with larger international FIT/FIT‐positive screening studies [12, 13]. Comparable studies have also shown a high prevalence of adenomatous and neoplastic lesions following positive stool‐based screening, supporting the necessity of colonoscopic assessment in these patients [6, 7, 8]. The dominance of neoplastic polyps in our participants is considered the most robust finding of the study. The reported frequency did not differ from that reported in a larger cohort, although the absolute number of colorectal cancer cases was small [12, 13]. These findings support, with caution due to the small sample size, the role of FIT as an effective screening tool for detecting patients at risk for colorectal neoplasia. Polyps were the most frequently identified colonoscopic finding. This finding was consistent with prior published literature showing that adenomatous polyps are among the most common lesions found during colonoscopy in individuals who test positive on FIT [6, 7, 8, 14]. Given that adenomatous polyps are recognized as precursors to colorectal cancer, their detection and excision are essential components of colorectal cancer prevention strategies [1, 2, 3, 4, 5, 15]. Furthermore, recent consensus guidelines advocate colonoscopic investigations following FIT positivity due to the increased likelihood of clinically significant colorectal lesions [5]. Hence, the relatively high prevalence of neoplastic polyps observed in this study is consistent with the clinical role of FIT as a triage tool for identifying patients who require colonoscopic evaluation.

In Lebanon, data on histopathological outcomes following positive FIT are still scarce, with most of the neoplastic polyps among FIT‐positive patients reported from European and Asian populations. Many factors may influence the diagnostic yield of FIT across different populations, such as healthcare access, patient referral patterns, screening uptake, and disease presentation. In Spain, Del Val Oliver et al. reported polyps in 203 patients, while only 2.5% had a normal colonoscopy [6]. In Turkey, Yücel et al. reported adenomatous polyps in 36.3% and adenocarcinoma in 9.7% of individuals [7]. Similarly, in Bangladesh, Siddiquee et al. reported polyps in 50.0% and a normal colonoscopy in 14.3% of patients [8]. Our findings are important to evaluate the reflection of the international screening recommendations in routine clinical practice in the Lebanese healthcare context. The 10.5% frequency observed in our study was similar to the 9.7% observed in Turkey. However, this similarity should be interpreted considering the differences in study design and patient selection. In our study, we included only FIT‐positive patients with abnormal colonoscopy findings and available histopathological results; thus, the high frequency of polyps and neoplastic lesions observed may not suggest an overall diagnostic yield of FIT among FIT‐positive individuals. Nevertheless, our findings may have particular importance to symptomatic patients, as a high proportion of our cohort who underwent FIT had gastrointestinal symptoms. FIT may potentially serve as a triage tool to prioritize symptomatic patients for colonoscopic evaluation, which differs from its established use for colorectal cancer screening in average‐risk asymptomatic patients [5]. Further prospective studies are needed, including all FIT‐positive patients irrespective of the colonoscopic findings.

Our findings reported that patients aged 45 years and older have a higher frequency of polyps than younger patients; however, these results need to be interpreted with caution, as patients under the age of 45 years represented a small proportion of the sample. Nevertheless, this result is consistent with available epidemiological information, which shows an increase in the incidence of colorectal neoplasia with increasing age [1, 2, 3]. Current colorectal cancer screening guidelines increasingly recommend initiating screening at the age of 45 due to the rising incidence of colorectal neoplasia in middle‐aged adults [5, 16, 17]. Consequently, the association between increased age and polyp identification found in this study further supports current screening recommendations and highlights the importance of age‐based risk stratification. However, larger cohorts with adequate representation of younger patients are needed to confirm this observation.

Schult et al. showed that FIT‐positive colonoscopy diagnostic yield is influenced by screening and patient personal characteristics, and confirmed that the most predominant findings among individuals with positive stool screening tests [12]. Selby et al. confirmed in their comprehensive review the importance of FIT‐based screening in identifying advanced and early‐stage colorectal cancer in large population settings [13]. These studies support the established role of FIT as a triage tool to identify patients who require colonoscopic evaluation. This potential application requires confirmation in larger prospective studies specifically designed to evaluate FIT in symptomatic populations.

Interestingly, our findings suggest that inflammatory lesions were more frequent in younger‐aged patients, taking into consideration the limited number of younger patients. In our dataset, inflammatory lesions were classified into a broader category including both ileitis and colitis, thus limiting the ability to distinguish between these specific conditions. Further larger studies are crucial to differentiate between the different inflammatory subtypes.

This distinction is relevant because recent evidence indicates that routine terminal ileal intubation in asymptomatic individuals undergoing colorectal cancer screening or post‐polypectomy surveillance has a low diagnostic yield. A recent systematic review and meta‐analysis reported a pooled yield of 1.74% for any ileal abnormality, 0.28% for clinically significant pathology, and 0.10% for Crohn's disease, suggesting that routine ileoscopy may not be warranted in asymptomatic screening populations [18]. However, these findings should be interpreted cautiously in relation to our cohort, which included both screening and symptom‐driven colonoscopies, particularly patients presenting with anemia, diarrhea, and abdominal pain. Therefore, our data do not permit conclusions regarding the value of routine ileoscopy, and future studies should distinguish isolated ileitis from colonic inflammatory lesions and assess its diagnostic relevance according to the indication for colonoscopy.

No significant association was observed between gender and clinical indications, colonoscopic findings, or histopathological outcomes in the present study. Although some previous studies reported higher rates of colorectal neoplasia among males, other studies demonstrated no significant sex‐based differences in diagnostic yield among FIT‐positive patients [7, 10, 11]. The absence of this association in our study may be related to the relatively limited sample size and single‐center design.

Broadly consistent with the age‐related patterns observed in our cohort, a Lebanese retrospective study reported that colorectal cancer was most prevalent among individuals aged 50–70 years of patients undergoing colonoscopy, however, inflammatory bowel disease was more common in younger patients aged 20–40 years [15].

The association between clinical indications and histopathological outcomes provided additional clinically relevant insights. Patients presenting with abdominal pain were more frequently represented in the non‐neoplastic lesion group compared with the neoplastic and malignant groups. Abdominal pain was significantly associated with non‐neoplastic histopathological findings, yet this association alone cannot be sufficient to discriminate for clinical prioritization of colonoscopy. Further prospective studies should assess whether clinical symptoms combined with FIT findings can enhance risk stratification and help in prioritization for colonoscopy.

In contrast, diarrhea demonstrated a statistically significant association with histopathological outcomes. Patients presenting with diarrhea were represented across non‐neoplastic, neoplastic, and malignant histopathological categories. These findings suggest that altered bowel habits in FIT‐positive patients may be associated with a broader spectrum of colorectal pathology. Previous studies have similarly reported associations between gastrointestinal symptoms, including altered bowel habits and diarrhea, and significant colonoscopic findings [9, 10, 11].

Neoplastic lesions were identified in 78.1% of patients scheduled for colonoscopy and 83.6% of patients tested for anemia. Although it was more prevalent in patients with anemia, the difference did not reach statistical significance.

This study has several limitations that should be acknowledged. Only patients with positive FIT results who underwent colonoscopy with abnormal findings and had available histopathological data were included in the study, which may introduce a selection bias by increasing the prevalence of neoplastic lesions, thus, limiting the generalizability of the findings to the overall population of FIT‐positive individuals Second, the retrospective observational nature of the study limits the ability to establish causal relationships between clinical indications and histopathological outcomes. Third, the study was conducted at a single center, which may limit the generalizability of the findings to broader populations. Fourth, the limited number of younger patients and malignant cases might have restricted the statistical power for the subgroup analyses. In addition, lifestyle factors, family history, use of medications, and other possible confounding factors were not studied.

Despite these limitations, the findings from this study add to the available literature regarding colonoscopic and histopathological outcomes in FIT‐positive patients. The relatively high prevalence of neoplastic and malignant lesions identified in this study emphasizes the importance of adherence to colonoscopic evaluation following positive FIT results. Recommended future multicenter prospective studies with larger samples are needed to further assess predictors of neoplastic and malignant colorectal lesions and to elucidate the role of clinical indications in colon cancer risk stratification.

## 5 Conclusion

In summary, neoplastic polyps represented the predominant histopathological finding in patients with positive fecal occult blood test (FIT) results and abnormal colonoscopy results, while a significant number of patients were found to have colorectal cancer. While the number of patients below 45 years of age was limiting, polyp frequency in older patients appeared substantially higher than in the younger group. These findings support the importance of timely colonoscopy evaluation following a positive FIT, particularly in patients presenting with higher‐risk clinical features. Our principal findings are consistent with international evidence, and this study contributes to the comprehensive real‐world descriptions of colonoscopic and histopathological outcomes among FIT‐positive patients internationally by including region‐specific evidence.

Additionally, this research offers important local information about histopathological results in FIT‐positive individuals in the Lebanese population. More extensive multicenter prospective studies are needed to better identify predictors of neoplastic and malignant lesions and to enhance risk stratification and colorectal cancer screening strategies.

## Author Contributions

Fatima El Houjeiry contributed to the conceptualization of the study, data collection, data curation, statistical analysis, interpretation of the data, and drafting of the manuscript. Walid Nasreddine contributed to the study methodology, supervision, interpretation of the findings, and critical revision of the manuscript. Alissar El Sibaii contributed to data collection, literature review, manuscript drafting, and manuscript revision. Hala Thaalibi contributed to data collection, data verification, and critical review of the manuscript. Bilal Azakir contributed to the conceptualization and design of the study, supervision of the research process, statistical oversight, interpretation of the results, critical revision of the manuscript, and final approval of the version to be published. All authors read and approved the final manuscript. Bilal Azakir had full access to all the data in the study and takes complete responsibility for the integrity of the data and the accuracy of the data analysis.

## Funding

The authors have nothing to report.

## Conflicts of Interest

The authors declare no conflicts of interest.

## Transparency Statement

The corresponding author (Bilal Azakir) affirms that this manuscript is an honest, accurate, and transparent account of the study being reported; that no important aspects of the study have been omitted; and that any discrepancies from the study as planned (and, if relevant, registered) have been explained.

## Data Availability

Data are available from Makassed General Hospital. Due to institutional and ethical restrictions related to patient confidentiality, the data are not publicly available. De‐identified data may be made available from the corresponding author upon reasonable request and with permission from Makassed General Hospital and the Institutional Review Board.
